# Clonal Dissemination of Plasmid-Mediated Carbapenem and Colistin Resistance in Refugees Living in Overcrowded Camps in North Lebanon

**DOI:** 10.3390/antibiotics10121478

**Published:** 2021-12-02

**Authors:** Adel Azour, Charbel Al-Bayssari, Tania Nawfal Dagher, Faraj Fajloun, Mark Fajloun, Jean-Marc Rolain

**Affiliations:** 1 Faculty of Sciences 3, Lebanese University, Michel Slayman Tripoli Campus, Ras Maska 1352, Lebanon; adelazour3@gmail.com (A.A.); faraj_fajloun@hotmail.com (F.F.); mark.fajloun1999@gmail.com (M.F.); 2 IRD, APHM, MEPHI, IHU-Méditerranée Infection, Aix Marseille Univ., 13005 Marseille, France; tania_nawfal28@hotmail.com; 3 Faculté de Médecine et de Pharmacie, 19-21 Boulevard Jean Moulin, CEDEX 05, 13385 Marseille, France; 4 Genomic Surveillance and Biotherapy Laboratory, Faculty of Sciences, Lebanese University, Ras Maska 1352, Lebanon

**Keywords:** refugees, *Enterobacteriaceae*, NDM-1, *mcr-1*

## Abstract

Carbapenem and colistin-resistant bacteria represent a global public health problem. Refugees carrying these bacteria and living in inadequate shelters can spread these microorganisms. The aim of this study was to investigate the intestinal carriage of these bacteria in Syrian refugees in Lebanon. Between June and July 2019, 250 rectal swabs were collected from two refugee camps in North Lebanon. Swabs were cultured on different selective media. Antibiotic susceptibility testing was performed using the disk diffusion method. Carbapenemase-encoding genes and *mcr* genes were investigated using real-time polymerase chain reaction (RT-PCR) and standard polymerase chain reaction (PCR). Epidemiological relatedness was studied using multilocus sequence typing (MLST). From 250 rectal swabs, 16 carbapenem-resistant, 5 colistin-resistant, and 4 colistin and carbapenem-resistant *Enterobacteriaceae* were isolated. The isolates exhibited multidrug-resistant phenotypes. Seven *Klebsiella pneumoniae* isolates harboured the *bla*_OXA-48_ gene, and in addition four *K. pneumoniae* had mutations in the two component systems pmrA/pmrB, phoP/phoQ and co-harboured the *bla*_NDM-1_ gene. Moreover, the *bla*_NDM-1_ gene was detected in six *Escherichia coli* and three *Enterobacter cloacae* isolates. The remaining five *E. coli* isolates harboured the *mcr-1* gene. MLST results showed several sequence types, with a remarkable clonal dissemination. An urgent strategy needs to be adopted in order to avoid the spread of such resistance in highly crowded underserved communities.

## 1. Introduction

Antimicrobial resistance (AMR) has increased markedly in recent years. This is particularly important when facing complex infections in humans [1]. AMR is expected to result in severe mortality and economic losses that will increase the cycle of poverty [2]. This global public health concern causes huge clinical and economical losses, mainly in developing countries [3]. Thus, the Centers for Disease Control and Prevention (CDC) and the World Health Organization (WHO) have recognised AMR as an urgent and global crisis and threat [4,5].

Carbapenems are a group of β-lactam drugs that are often used as last resort antibiotics to treat infections arising from multidrug-resistant (MDR) Gram-negative bacteria [6]. Unfortunately, carbapenemase-producing Gram negative bacteria have become a major concern around the world [7,8,9]. They are a growing concern because they confer resistance to β-lactam antibiotics and other classes of antibiotics such as aminoglycosides, fluoroquinolones, and cotrimoxazole [10]. This reduces the possibility of treating infections due to such multi-resistant organisms [11].

Currently, carbapenemases in *Enterobacteriaceae* are found mainly in *Klebsiella pneumoniae*, *E. coli*, and other enterobacterial species, with a greater prevalence in southern Europe and Asia than in other parts of the word [6]. New Delhi metallo-β-lactamase-1 (NDM-1) is the most frequent carbapenemase found in Asia [12]. A recent study conducted in Nepal in 2019 [13] showed that the prevalence of MDR bacteria was 58% (199/343 isolates), with a prevalence of 12.5% for carbapenemase-producing bacteria and 3% for colistin-resistant bacteria. Interestingly, a study published in 2021 [14] in the same country, showed the rapid dissemination of carbapenem-resistant bacteria. From a total of 58 *K. pneumoniae* isolates, 60% (35/55) were carbapenem resistant with NDM-1 being the most dominant carbapenemase (80%).

On the other hand, *K. pneumoniae* carbapenemase (KPC) is mainly found in the United States and in Europe (France, Greece, and Italy) [12]. Several studies have reported an increased spread of KPC-producing *K. pneumoniae* such as the recent study conducted in Italy where 50% of carbapenem-resistant isolates were KPC-2 producers [15]. Moreover, OXA-48 carbapenemase is the main carbapenemase found in Turkey, Malta, and the Middle East [12]. Following its initial description in Turkey, OXA-48 carbapenemase has been reported in different parts of the world, mainly in the Mediterranean basin where several studies have described the emergence and spread of OXA-48 producing bacteria in that region [16].

Moreover, the emergence of MDR and extensively-drug-resistant (XDR) Gram-negative bacteria, as well as the lack of new agents against these pathogens, led to the reintroduction of colistin [17]. The use of colistin had been abandoned many years ago due to concerns about nephrotoxicity, however it is now regarded as a last resort option in infections caused by MDR bacteria [11,18,19,20]. The mechanisms of colistin resistance are complex and varied and can range from chromosomal to plasmidic. In addition, there is also a type of adaptive resistance that is simply related to the modification of lipopolysaccharides (LPS) moiety that changes the charge of the molecule, thus preventing binding [21,22,23].

The population of Lebanon was estimated to be 3,759,136 in 2007. This number increased to 4,822,000 in 2013, of which 21% were under 15 years of age and 12% over 60 years of age [24].

This dramatic increase is partly attributed to the influx of Syrian refugees following the declaration of war in Syria in 2012, which caused a large budget deficit exacerbated by the withdrawal of international organisations that had promised to participate in funding [25]. Many Syrian refugees currently in Lebanon live in poor living conditions with limited access to healthcare, clean water, and inadequate food [26,27].There is no reliable health-related database to promote better care for this underserved population. More importantly, there is little data available concerning the epidemiology of antimicrobial resistance in the Syrian refugee population. The gastrointestinal tract is known to be a reservoir of Gram negative *Enterobacteriaceae*, and it has been shown that colonisation by MDR *Enterobacteriaceae* increase by at least two-fold the risk of subsequent infection [28]. Given that this may cause barriers to primary health care, we attempted to look at the prevalence of antimicrobial resistance, in particular carbapenem and colistin resistance genes which were isolated from bacteria, particularly *Enterobacteriaceae*, among Syrian refugees in North Lebanon, who live in unhygienic conditions, have limited toilet facilities, consume polluted water, very poor food quality, and live in overcrowded environments.

## 2. Materials and Methods

### 2.1. Study Design

**Figure 1:**
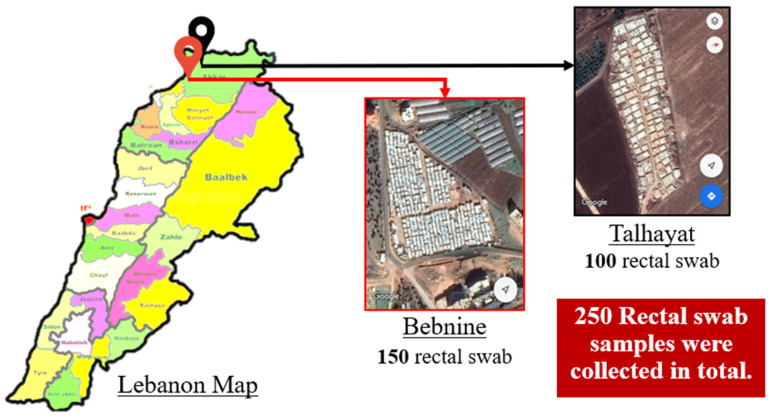
The location of the two camps in Lebanon where samples were taken.

Between June and July 2019, 250 rectal swabs were collected from the inhabitants of two Syrian refugee camps, located in North Lebanon, in the Akkar Governorate (Figure 1). One hundred random samples were collected from the Talhayat camp and another 150 from the Bebnine camp. The swabs were grouped according to whether they came from residents of the same tent and/or family. The swabs were inserted through the rectal sphincter 1–1.5 inches (2–3 cm) and rotated gently. Tubes containing the swabs were placed in a refrigerator (2–8 °C), after checking that faecal material was visible on the tip of the swab. The swabs were directly shipped to the laboratory (less than 1.5 h away) where they were directly cultivated on Tryptic soy broth (TSB) medium (Becton Dickinson GmbH, Heidelberg, Germany).

### 2.2. Bacterial Identification

The rectal swabs were cultivated in the growth medium Tryptic soy broth (TSB) (Becton Dickinson GmbH, Heidelberg, Germany) and incubated for 24 h at 37 °C. Different selective media, including Lucie Bardet Jean-Marc Rolain (LBJMR) [29] and MacConkey agar (bioMérieux, Marcy l’Etoile, France) supplemented with Ertapenem (2 μg/mL) were then used to culture 100 μL of the enrichment medium. These media were used to screen colistin resistant and carbapenem resistant organisms, respectively. The selected isolates were then sent to the laboratory in Marseille, France. Bacterial identification at the species level was performed using matrix-assisted laser desorption/ionization time-of-flight mass spectrometry (MALDI-TOF) (Microflex; Bruker Daltonics).

### 2.3. Antibiotic Susceptibility Profile

The antibiotic susceptibility profile of the isolates was identified using the standard disk diffusion method on Mueller–Hinton agar (bioMérieux, Marcy l’Etoile, France). Sixteen different antibiotics were tested, including amoxicillin (20 μg), amoxicillin-clavulanic acid (20/10 μg), piperacillin-tazobactam (30/6 μg), cephalotin (30 μg), ceftriaxone (30 μg), cefepime (30 μg), ertapenem (10 μg), imipenem (10 μg), amikacin (30 μg), gentamicin (10 μg), ciprofloxacin (5 μg), Fosfomycin (200 μg), nitrofurantoin (100 μg), tobramycin (10 μg), trimethoprim-sulfamethoxazole (1.25/23.75 μg), and colistin (10 μg) (bioMérieux, Marcy l’Etoile, France). The minimal inhibitory concentration (MIC) of colistin, ertapenem, and imipenem was identified using the microdilution and the E-test methods, respectively. Each strain was considered to be resistant to colistin, ertapenem, and imipenem if their MICs were greater than 2 mg/L, 1 mg/L, and 8 mg/L, respectively. The results were interpreted according to the European Microbial Medical Sensitivity Committee (EUCAST) 2017 (http://www.Sfmicrobiology.Org/Userfiles/Files/Files/CASFM/CASF%20V2_0_MAI2017.PDF, accessed on 25 September 2020).

### 2.4. Phenotypic Detection of Carbapenemase Activity

Three phenotypic tests, the modified Hodge test (MHT), the modified Carba NP test (MCNP), and the ethylene diamine tetra-acetic acid (EDTA) test, were performed to detect the production of carbapenemase, as described previously [30,31].

### 2.5. DNA Extraction

The extraction of the bacterial DNA was performed by the EZ1-automatic robot (Qiagen Biorobot EZ1-, Tokyo, Japan) using an extraction kit (DNA EZ1, Qiagen, Hilden, Germany), following the manufacturer’s instructions. The extracted DNA was kept at −20 °C. In order to determine the genetic location of the carbapenemase and *mcr* genes, plasmid extraction was performed for the isolates that showed a positive PCR result for these genes using the GeneJET Plasmid Miniprep Kit (Thermo Fisher Scientific, Waltham, MA, USA).

### 2.6. Molecular Identification of Carbapenem and Colistin Resistance Genes

The presence of carbapenemase encoding genes such as *bla*_OXA-48_, *bla*_OXA-58_, *bla*_KPC_, *bla*_NDM_, and *bla*_VIM_ was investigated by the real-time PCR assay (RT-PCR) (Bio-Rad, CFX96 Touch^TM^, Hercules, CA, USA) using specific primers, as previously described [32,33]. Moreover, RT-PCR for colistin resistant isolates was performed to detect the presence of *mcr-1*, *mcr-2*, *mcr-3*, *mcr-4*, *mcr-5*, and *mcr-8* genes using previously described primers and probes [34,35].

Standard PCR and sequencing of antibiotic resistance genes was performed on an automated ABI 3130 sequencer (PE Applied Biosystems, Foster City, CA, USA) using BigDye terminator chemistry. The sequenced genes were analysed using BlastN and BlastP and then compared with the ARG-ANNOT database.

All mcr-negative colistin-resistant bacteria were investigated for the presence of possible mutations in colistin-resistance genes such as *pmrA, pmrB*, *phoP*, *phoQ*, and *mgrB* by amplification and sequencing [32,36,37]. Those genes were compared with reference strains such as *E. coli* K-12 MG 1655 (NCBI GenBank accession no. U00096.3), *K. pneumoniae* MGH 78578 (NCBI GenBank accession no. CP000647), and *E. cloacae* ATCC 13047 (NCBI GenBank accession no. CP001918.1) using nps alignment software (https://prabi.ibcp.fr/htm/site/web/home, accessed on 18 February 2021).

In order to determine whether the recognised amino acid substitution resulting from a missense mutation could affect the function of the protein, PROVEAN (Protein Variation Effect Analyser) software (http://provean.jcvi.org/seq_submit.php, accessed on 18 February 2021) was used. If the score of the variant proteins was below or equal to a predefined threshold (−2.5), it was considered to have a “deleterious” effect, and if the score was above the threshold, it was predicted to have a “neutral” effect.

### 2.7. Multilocus Sequence Typing (MLST)

In order to determine the clonal relationship of the isolates, the MLST method was performed as described on the Institute Pasteur’s MLST website (www.pasteur.fr/mlst, accessed on 23 March 2021).

## 3. Results

From 250 rectal swabs, 25 carbapenem and/or colistin resistant strains were isolated (10%). Among the isolates, 16 carbapenem-resistant strains were identified, namely, seven *Klebsiella pneumoniae*, six *Escherichia coli*, and three *Enterobacter cloacae*. Moreover, five *E. coli* isolates were resistant to colistin. The remaining four isolates were identified as *K. pneumoniae* and were resistant to carbapenem and colistin. The antibiotic resistance profile is presented in Table 1 for carbapenem-resistant (CarbaR) strains and in Table 2 for colistin-resistant (ColiR) strains. Imipenem MICs ranged from 2 to >32 mg/L (Table 1 and Table 2) and ertapenem MICs ranged from 4 to >32 mg/L (Table 1 and Table 2). In contrast, according to the Unitary Minimum Inhibitory Concentration (UMIC) test, colistin MICs for *K. pneumoniae* and *E. coli* isolates were >64 mg/L and 4 mg/L, respectively (Table 2). The MCNP test and the MHT were positive for all carbapenem-resistant isolates, whereas the EDTA test was positive for all carbapenem-resistant isolates except for KP-2, KP-3, KP-4, and KP-5 isolates. Real-time PCR and standard PCR results showed that *E. coli* CarbaR (EC-1, EC-2, EC-3, EC-4, EC-5, and EC-6), *E. cloacae* CarbaR (Eclo-1, Eclo-2, and Eclo-3), and *K. pneumoniae* ColiR (KP-1, KP-2, KP-3, and KP-4) were positive for *bla*_NDM_ gene. Sequence analysis of detected carbapenemases identified the *bla*_NDM-1_ gene in positive strains (Table 1 and Table 2). In contrast, results showed that *K. pneumoniae* CarbaR (KP-2, KP-3, KP-4, KP-5, KP-6, KP-7, and KP-8) were positive for the *bla*_OXA_ gene. Sequence analysis of detected carbapenemases identified the *bla*_OXA-48_ gene in positive strains (Table 1). Moreover, results showed that *K. pneumoniae* CarbaR strains (KP-6, KP-7, and KP-8) harboured, in addition, the *bla*_NDM_ gene. Sequence analysis of detected carbapenemases identified the *bla*_NDM-1_ gene in positive strains (Table 1). In addition, results showed that *E. coli* coliR (EC-7, EC-8, EC-9, EC-10, and EC-11) were positive for the *mcr-1* gene and were identified as the *mcr-1* gene by sequencing (Table 2).

Moreover, PCRs performed on extracted plasmids (from isolates that had a positive PCR for carbapenemase and *mcr-1* genes), showed a positive result for these genes assuming a plasmidic localisation.

Due to the absence of *mcr* genes in *K. pneumoniae* ColiR (Table 2), genes implicated in colistin resistance (*mgrB*, *pmrA*, *pmrB*, *phoP*, and *phoQ*) were amplified and sequenced. As shown in Table 3, the result of the sequence analysis showed that there was no mutation in the *mgrb*, *pmrA*, and *phoP* genes for the four colistin-resistant *K. pneumoniae* isolates. These isolates were resistant to colistin due to mutations in the *pmrB* and *phoQ* genes (Table 3). For the colistin resistant Kp-1 isolate, the analysis revealed a nucleotide deletion (C577del) in the *pmrB* gene, leading to a frameshift mutation and resulting in a defective protein. For the colistin resistant Kp-2, the analysis revealed a nucleotide insertion (G432_C433insG) in the *pmrB* gene, causing a frameshift mutation and resulting in a defective protein. For the Kp-3 isolate, the analysis also revealed a nucleotide insertion at position (T459_G460insC) in the *phoQ* gene leading to a frameshift mutation and resulting in a defective protein. Finally, for the Kp-4 isolate, the analysis showed two genetic modifications: a nucleotide insertion (G623_C624insG) in the *pmrB* gene leading to a frameshift mutation resulting in a defective protein, and a nucleotide insertion at position (T435_A436insT) in the *phoQ* gene causing a frameshift mutation resulting in a defective protein.

MLST analysis revealed that nine *E. coli* isolates belonged to six different sequence types (STs) including ST361 (*E. coli* carbaR: EC-1, EC-2, EC-3), ST1294 (*E. coli* carbaR: EC-4, EC-5), ST648 (*E. coli* CarbaR: EC-6) (Table 1), ST2001 (*E. coli* ColiR: EC-7, EC-8, EC-9), ST101 (*E. coli* ColiR: EC-10), and ST4187 (*E. coli* ColiR: EC-11). *K. pneumoniae* isolates had several STs such as ST14 (*K*. *pneumoniae* CarbaR: KP-2, KP-3, KP-4, Kp-5), ST16 (*K*. *pneumoniae* CarbaR: KP-6, KP-7, KP-8) (Table 1), and ST944 (KP-1, KP-2, KP-3, KP-4) (Table 2), and the tree *E. cloacae* belonged to ST182 (Eclo-1, Eclo-2) and ST1120 (Eclo-3) (Table 1).

## 4. Discussion and Conclusions

MDR bacteria are now considered as a serious public health problem due to the limited range of antibiotics used to treat infections caused by these bacteria. The antimicrobial resistance crisis is mainly due to the misuse of these antibiotics in several sectors (human and animal) and the luck of discovery of new drugs [38]. Refugees coming from countries with a high occurrence of antimicrobial resistance can introduce the resistant isolates to their new country of residence. These refugees can spread these resistant bacteria, especially if they are living in shelters with poor living conditions. This study describes the intestinal carriage of carbapenem- and colistin-resistant bacteria particularly in *Enterobacteriaceae* among healthy Syrian refugees living in two shelters (Bebnine and Talhayat) in North Lebanon.

In this study, 25 carbapenem- and/or colistin-resistant isolates were collected from 250 rectal swabs isolated from healthy Syrian refugees. All isolates were resistant to the majority of antibiotics tested. PCR and sequencing showed the presence of 16 carbapenem-resistant isolates (6 *E. coli*, 7 *K. pneumoniae*, and 3 *E. cloacae*), 5 colistin-resistant isolates (*E. coli*), and 4 carbapenem and colistin-resistant isolates (*K. pneumoniae*) (Table 1 and Table 2).

All carbapenem-resistant *E. coli* isolates (six isolates) harboured the *bla*_NDM-1_ gene. Five of them (EC-1, EC-2, EC-3, EC-4, and EC-5) were collected from the same shelter (Bebnine), and from the same tent; three isolates (EC-1, EC-2, and EC-3) belonged to ST361 and two (EC-4 and EC-5) belonged to ST1294. The *bla*_NDM-1_ *E. coli* ST361 had already been reported in hospitalised patients in South Korea [39]; however, ST1294 is mainly found in animals and in the environment [40,41,42,43,44,45]. These results shed light on the clonal dissemination of these isolates between refugees, since the same clones were found in more than one person in the same tent, but also to the probable zoonotic or environmental origin of the ST1294 isolates. The sixth isolate (EC-6) belonged to ST648 and was collected from the Talhayat shelter. This clone was previously reported in different studies in hospitalised patients [46,47,48]. In Lebanon, NDM-1 producing *E. coli* isolates were first described in 2012 in Iraqi patients referred to Lebanon [49]. Moreover, NDM-1 producing *E. coli* was also reported in 2013 at a tertiary care centre in Lebanon [50]. To the best of our knowledge, this is the first report of intestinal carriage of NDM-1 producing *E. coli* in healthy individuals.

All carbapenem-resistant *K. pneumoniae* isolates (seven isolates: KP-2, KP-3, KP-4, KP-5, KP-6, KP-7, and KP-8) harboured the *bla*_OXA-48_ gene, of which three also had the *bla*_NDM-1_ gene (KP-6, KP-7, and KP-8). The carbapenem-resistant *K. pneumoniae* isolates (KP-2, KP-3, KP-4, and KP-5) harbouring the *OXA-48* gene had the same ST (ST16) and were collected from the same shelter (Bebnine) and from closely related tents. OXA-48 producing *K. pneumoniae* ST16 was previously described in clinical isolates in Spain [51]. In Lebanon, several studies have reported the occurrence of OXA-48 producing bacteria and OXA-48 producing *K. pneumoniae*, all from hospital settings [49,52,53,54]. In addition, the remaining three isolates (KP-6, KP-7, and KP-8) harbouring both the *NDM-1* and the *OXA-48* genes, also had the same ST (ST14), and were collected from the Talhayat shelter from closely related tents. NDM-1 and/or OXA-48 producing *K. pneumoniae* ST14 have been reported worldwide [55,56,57,58], highlighting the rapid spread of this clone. In Lebanon, several studies have described NDM-1 and/or OXA-48 producing *K. pneumoniae* isolates [49,52,53]; however, this is the first description of the co-occurrence of *NDM-1* and *OXA-48* genes in the same isolate, which highlights the dissemination of plasmids carrying these resistance genes between isolates. Furthermore, results showed that *K. pneumoniae* isolates recovered from closely related tents in the same shelter belonged to the same clone. Syrian refugees in Lebanon live in inadequate shelters living in crowded conditions with poor sanitary facilities, which increases the spread of resistant bacteria. This explains the dissemination of the same clone in refugees living in closely related tents within the same shelter.

All carbapenem-resistant *E. cloacae* isolates (Eclo-1, Eclo-2, Eclo-3) harboured the *bla*_NDM-1_ gene, of which two (Eclo-1 and Eclo-2) were isolated from the same tent at the Bebnine shelter and belonged to the same clone (ST182). The remaining isolate belonged to ST1120 and was isolated from the Talhayat shelter. NDM-1 producing *E. cloacae* ST182 have been reported in many countries, including China, Czech Republic, and Kenya [59,60]. This clone is of high concern because of its ability to disseminate rapidly, causing outbreaks as was the case in Mexico [61]. Hence, controls should be performed to avoid the rapid dissemination of such clones. To the best of our knowledge, this is the first detection of NDM-1 producing *E. cloacae* in Lebanon.

In total, nine colistin-resistant isolates were collected, five *E. coli* (EC-7, EC-8, EC-9, EC-10, and EC-11) and four *K. pneumoniae* (ColiR KP-1, ColiR KP-2, ColiR KP-3, and ColiR KP-4).

All colistin-resistant *E. coli* isolates harboured the *mcr-1* gene, of which three (EC-7, EC-8, EC-9) belonged to the same clone ST2001 and were isolated from closely related tents at the Bebnine shelter. The remaining *E. coli* isolates belonged to ST101 (Bebnine shelter) and ST4187 (Talhayat shelter). *E. coli* ST2001 has been previously described in feacal samples from wild animals [62] and in an organic broiler farm [63]. ST4187 has been described in broiler chickens in Tunisia [64], thus pointing towards a zoonotic origin of our isolates, since the refugees are in close contact with wild animals. The ST101 clone has been reported in clinical samples from Brazil and Iran [65,66]. In Lebanon, *E. coli* harbouring the *mcr-1* gene has been described in few studies in hospitalised patients [67], however, no other study has highlighted its presence in healthy individuals. To the best of our knowledge, this is the first description in Lebanon of the intestinal carriage of *E. coli* isolates harbouring the *mcr-1* gene in healthy individuals. This is alarming, since the plasmid carrying the *mcr-1* gene could disseminate between species, making them resistant to one of our last resort antibiotics.

All colistin-resistant *K. pneumoniae* isolates harboured the *bla*_NDM-1_ gene and had mutations in the *PmrB* and/or *PhoQ* genes (Table 3). They were all isolated from the same family (in the Bebnine camp) and belonged to the same clone ST944. An ST944 clone harbouring the *mcr-1* gene has been previously described in chicken meat from western Algeria [68]. A recent study conducted in Egypt [69] described colistin and carbapenem resistance in *K. pneumoniae* strains isolated from chicken and humans. The study showed that all *K. pneumoniae* were MDR, with 45.9% harbouring a ß-lactamase gene with a carbapenemase gene, 18.9% harbouring a ß-lactamase gene with the *mcr-1* gene, and 13.5% harbouring a β-lactamase gene with both carbapenemase and *mcr-1* genes. In Lebanon, few studies have described colistin-resistance in *K. pneumoniae* isolates such as those in 2015 [70] and 2017 [71]. Our study showed new mutations (Table 3) in the *pmrB* and/or *phoQ* genes resulting in a defective protein with a stop codon. Interestingly, *a* study conducted in Lebanon in 2018 [72] described colistin-resistance in clinical *K. pneumoniae* isolates due to mutations in the *mgrB*, *pmrB*, and *phoQ* genes. These isolates also harboured the *bla*_NDM-5_ gene. Our study reported for the first time in Lebanon, colistin-resistance *K. pneumoniae* isolates with new mutations in *pmrB* and/or *phoQ* genes and harbouring the *bla*_NDM-1_ gene.

In conclusion, the intestinal carriage of MDR bacteria in community settings is of great concern. Our study reported for the first time the intestinal carriage of carbapenem- and/or colistin-resistant isolates in refugees, which is alarming since plasmids carrying resistance genes can spread between bacteria and refugees within the same shelter. Syrian refugees come from a country with a high prevalence of antimicrobial resistance where antibiotics can be taken without prescription, thus they can spread MDR bacteria to their final destination, in this case, Lebanon. In addition, these refugees are living in inadequate shelters which can contribute to the spread of these bacteria. Molecular and epidemiological studies are essential in order to better understand the mode of transmission of these microorganisms. An urgent strategy must therefore be adopted to limit the spread of these MDR bacteria within the community.

## Figures and Tables

**Table 1:** Phenotypic and genotypic features of the carbapenem-resistant *Enterobacteriaceae* isolates.

	Strain Name	Source	Antibiotic Susceptibility Profile	MAR Index	IMP MIC (μg/mL)	ERT MIC (μg/mL)	Carba NP Test	*bla*_OXA-48_	*bla*_NDM-1_	ST	Accession Number
Same tent (same family)	*E. coli* EC-1	BN	FF, F, AK, CS, CN	0.68	12	>32	+	−	+	361	OL542491
	*E. coli* EC-2	BN	FF, F, AK, CS, CN	0.68	>32	>32	+	−	+	361	OL542492
	*E. coli* EC-3	BN	FF, F, AK, CS, CN	0.68	16	>32	+	−	+	361	OL542493
	*E. coli* EC-4	BN	FF, F, AK, CS, CN	0.68	>32	>32	+	−	+	1294	OL542494
	*E. coli* EC-5	BN	FF, F, AK, CS, CN	0.68	>32	>32	+	−	+	1294	OL542495
	*E. coli* EC-6	TL	FF, F, AK, CS, CN	0.68	4	>32	+	−	+	648	OL542496
Same tent (same family)	*E. cloacae* Eclo-1	BN	DO, CS	0.87	4	12	+	−	+	182	OL474360
	*E. cloacae* Eclo-2	BN	CS	0.93	4	8	+	−	+	182	OL474361
	*E. cloacae* Eclo-3	TL	CS	0.93	4	8	+	−	+	1120	OL474362
Tents are close (Not the same family)	*K. pneumoniae* KP-2	BN	AK, CS, CN	0.81	>32	>32	+	+	−	16	OL542509
	*K. pneumoniae* KP-3	BN	AK, CS, CN	0.81	>32	>32	+	+	−	16	OL542510
	*K. pneumoniae* KP-4	BN	TZP, CS	0.87	>32	>32	+	+	−	16	OL542511
	*K. pneumoniae* KP-5	BN	AK, CS, CN	0.81	>32	>32	+	+	−	16	OL542512
Tents are close (Not the same family)	*K. pneumoniae* KP-6	TL	DO, CS	0.87	>32	>32	+	+	+	14	OL542513 OL542506
	*K. pneumoniae* KP-7	TL	AK, DO, CS	0.81	>32	>32	+	+	+	14	OL542514 OL542507
	*K. pneumoniae* KP-8	TL	FF, AK, DO, CS	0.75	>32	>32	+	+	+	14	OL542515 OL542508

Fosfomycin (FF), nitrofurantoin (F), amikacin (AK), doxycycline (DO), colistin (CS), piperacillin-tazobactam (TZP), cefalotin (CF), gentamicin (CN), ertapenem (ERT), imipenem (IMP), minimum inhibitory concentration (MIC), sequence type (ST), multiple antibiotic resistance (MAR), Talhayat (TL), and Bebnine (BN).

**Table 2:** Phenotypic and genotypic features of the colistin and carbapenem-resistant *Enterobacteriaceae* isolates.

	Strain Name	Source	Antibiotic Susceptibility Profile	MAR Index	IMP MIC (μg/mL)	ERT MIC (μg/mL)	UMIC Test (μg/mL)	Carba NP Test	*bla*_NDM-1_	*mcr-1*	Mutations of the Associated Colistin-Resistance Proteins	ST	Accession Number
Tents are close (Not the same family)	*E. coli* EC-7	BN	FEP, TZP, CRO, ERT, IMP, FF, F, AK, CN	0.43	−	−	4	−	−	+	−	2001	OL542497
	*E. coli* EC-8	BN	FEP, TPZ, CRO, ERT, IMP, FF, F, AK, CN, SXT	0.37	−	−	4	−	−	+	−	2001	OL542498
	*E. coli* EC-9	BN	FEP, TPZ, CRO, ERT, IMP, FF, F, AK, CN, SXT	0.37	−	−	4	−	−	+	−	2001	OL542499
	*E. coli* EC-10	BN	FEP, TPZ, CRO, ERT, IMP, FF, F, AK, CN	0.43	−	−	4	−	−	+	−	101	OL542500
	*E. coli* EC-11	TL	FEP, TPZ, CRO, ERT, IMP, FF, F, AK, CN, SXT	0.37	−	−	4	−	−	+	−	4187	OL542501
Different tents (Same family)	*K. pneumoniae ColiR* KP-1	BN	−	1	4	4	>64	+	+	−	+	944	OL542502
	*K. pneumoniae ColiR* KP-2	BN	−	1	2	4	>64	+	+	−	+	944	OL542503
	*K. pneumoniae ColiR* KP-3	BN	−	1	2	4	>64	+	+	−	+	944	OL542504
	*K. pneumoniae ColiR* KP-4	BN	−	1	2	4	>64	+	+	−	+	944	OL542505

Cefepime (FEP), piperacillin-tazobactam (TZP), ceftriaxone (CRO), ertapenem (ERT), imipenem (IMP), fosfomycin (FF), nitrofurantoin (F), amikacin (AK), gentamicin (CN), trimethoprim-sulfamethoxazole (SXT), doxycycline (DO), colistin (CS), minimum inhibitory concentration (MIC), sequence type (ST), multiple antibiotic resistance (MAR), Talhayat (TL), and Bebnine (BN).

**Table 3:** Results of the nucleotide mutations of the colistin-resistant strains.

Strain Name	Mgrb	PmrA	PmrB	PhoP	PhoQ	Accession Number
Coli R Kp 1	No mutation	No mutation	C577del	No mutation	No mutation	OL587685
Coli R Kp 2	No mutation	No mutation	G432_C433insG	No mutation	No mutation	OL587686
Coli R Kp 3	No mutation	No mutation	No mutation	No mutation	T459_G460insC	OL587683
Coli R Kp 4	No mutation	No mutation	G623_C624insG	No mutation	T435_A436insT	OL587687 OL587684

## Data Availability

The data presented in this study are available in Table 1 and Table 2.
